# Dynamic intrafractional position monitoring with implanted fiducial markers for enhanced accuracy in radiotherapy of prostate cancer

**DOI:** 10.1007/s13246-023-01304-w

**Published:** 2023-07-31

**Authors:** Julian Mangesius, Thomas Seppi, Ramine Ibrahim, Katrin Fleischmann, Angela Ginestet, Samuel Vorbach, Tilmann Hart, Martin Pointner, Stephanie Mangesius, Ute Ganswindt

**Affiliations:** 1 Department of Radiation Oncology, Medical University of Innsbruck, Univ.-Klinik für Strahlentherapie-Radioonkologie, Anichstrasse 35, Innsbruck, 6020 Austria; 2 Department of Neuroradiology, Medical University of Innsbruck, Innsbruck, Austria

**Keywords:** Prostate cancer, Stereotactic body radiation therapy, Intrafractional image guided radiotherapy, Intrafractional organ monitoring, Translational and rotational prostate motion, Exactrac dynamic system

## Abstract

Introduction: Recent advances in the radiation therapy of prostate cancer have brought a shift toward moderate- and ultra-hypofractionated treatment schedules. Reducing safety margins can broaden the therapeutic window in stereotactic treatments and alleviate concerns for toxicity in high dose-per-fraction treatment schedules. Management of intrafractional motion is a necessity for stereotactic body radiation therapy (SBRT). It can be achieved by performing intrafractional image guidance and position corrections. We evaluate the suitability of such a novel prostate motion management system and its potential benefit for treatment accuracy. Methods: Intrafractional IGRT was performed for 22 patients during 149 treatment sessions using repeated orthogonal kV-XR imaging of implanted fiducial markers with the ExacTrac Dynamic (EXTD) system. Position measurements were taken four times during each arc of the applied volumetric modulated arc therapy (VMAT). Position correction was performed if translational deviation exceeded 2 mm in any direction. Results: Of 677 single EXTD measurements, 20.6% exceeded the predefined threshold of 2 mm 3D deviation. Without intrafractional corrections, 39.4% of all individual measurements would exceed the threshold. The 3D accuracy could thus significantly be improved, reducing mean 3D shifts from 1.97 (± 1.44) mm to 1.39 (± 1.01) mm by performing intrafractional IGRT. In total, 34% of all treatment sessions required correction of intrafractional position shifts. Conclusion: Monitoring of prostate motion using repeated intrafractional orthogonal kV-X-ray-based position measurements of implanted fiducial markers proved to be a reliable method to improve precision of stereotactic irradiations of the prostate. It can prevent unacceptable translation deviations in one third of all sessions.

**Supplementary Information:**

The online version contains supplementary material available at 10.1007/s13246-023-01304-w.

## Introduction

With the aid of constantly increasing technical and computational advancement, substantial gains have been achieved during the last two decades in precise imaging, accurate contouring of target volumes, and in adequately accelerating complex and individualized treatment planning and dose delivery - thereby extending the therapeutic window in the curative setting [1]. Sources of persisting uncertainties highly depend on location of the target volume with the highest degree of planning and treatment inaccuracy among motion-afflicted target sites, such as the prostate [2, 3]. This organ is particularly prone to substantial position shifts and rotational dislocations between as well as within single treatment sessions of fractionated irradiation [4]. Moreover, prostate movements are unpredictable and dependent on random changes in bladder and rectal filling and involuntary tension or relaxation of muscles [5, 6]. This leads to translational and rotational organ motion, with anterior-posterior reported to be the main direction of translation, and pitch being the predominant direction of rotation [7]. With application of common safety margins and daily-performed imaging verification before treatment, acceptable accuracy with standard fractionation schemes can be ensured [8]. However, this setting might no longer warrant adequate dose delivery in more modern dose concepts of increasingly applied moderately hypofractionated, and especially, of ultra-hypofractionated schemes [9]. As the number of fractions decreases, the relevance of intrafractional random deviations becomes significant and it must result in the application of corrective measures. For this purpose, methods have been developed to monitor and correct for organ motion during treatment [10–12]. Suitable systems should allow for repeated non- or minimal-invasive intrafractional recording of 6D organ position (translation and rotation), as well as for automated beam-hold upon exceedance of predefined motion tolerances. Systems capable of real-time continuous tracking, as well as discontinuous repeated monitoring of motion are available [13, 14].

Accurate margin setup is imperative in optimized radiotherapy, since the toxicity of prostate irradiation is still a matter of concern, especially in the context of hypofractionated concepts. Increasing accuracy of treatment delivery should potentially allow for tighter margins of planning target volumes [8], thereby enabling better protection of surrounding organs at risk (OAR) without compromising tumour control [14, 15].

Herein we describe and evaluate a novel system using x-ray (XR)-based IGRT for various indications including a new capability for intrafractional prostate motion management. Particularly, the aim of the present study was to evaluate the feasibility of multiple intrafractional position measurements using the ExacTrac Dynamic System (EXTD) to monitor and correct prostate motion during high-precision radiotherapy. We compare the achievable accuracy of this system and its applicability for hypofractionation schedules and SBRT treatments to existing methods for intrafractional IGRT.

## Methods

Patients included in this analysis received primary radiation treatment for localized lymph node negative prostate cancer. Applied treatment schedules comprised conventional (n = 7) and moderately hypofractionated (n = 15) radiotherapy (RT) of the prostate and seminal vesicles if indicated. Conventionally fractionated RT was applied with a target dose of 76 Gy in 38 fractions, 5 times a week, and hypofractionated RT with 60 Gy in 20 fractions, 5 times a week. Treatment planning was performed using Pinnacle Software (V.14; Philips Medical, Fitchburg, USA) for VMAT-IMRT with 6MV Photons delivered in one full arc. Radiation treatment was performed using a Versa HD linear accelerator with the Agility collimator system (Elekta AB, Stockholm, Sweden) and a cone beam CT (CBCT). ExacTrac Dynamic (EXTD; V1.1; Brainlab, Munich, Germany) was employed for initial patient set-up as well as for intrafractional position measurements.

The ExacTrac Dynamic system consists of two kV X-ray (XR) (kV) tubes located in the floor and two flat panel detectors mounted diagonally each on the opposite side of the ceiling, with a field-of-view (FOV) of 18 × 18 cm at the isocentre for both panels. The XR system acquires stereoscopic images of anatomic structures focused on the machine isocentre, which are automatically fused to digitally reconstructed radiographs (DRRs) calculated from the planning CT. The calculated 6D positional shift can be used for both initial patient positioning and intrafractional monitoring. This setup also allows the user to monitor implanted fiducial markers. The system automatically detects the gold markers in the XR images and matches them to the expected position in the DRRs. The system can acquire stereoscopic images for full 6D position measurement only at the cardinal angles of 0°, 90°, 180°, and 270°, since the view is obstructed by the gantry at other angles. An example of the images acquired by the XR system is shown in the supplementary figure. In addition to the XR guidance, the system also incorporates a surface tracking module utilizing optical and thermal cameras, which is mounted on the ceiling in the centre position between the flat panel detectors. In combination with the XR based monitoring, this module can be used for surface guided pre-positioning and intrafractional surface tracking.

The EXTD system has been commissioned at our institution according to AAPM TG-302 [16] and the ESTRO-ACRPO guideline on surface guided RT (SGRT) [17] for all surface guided workflows. In addition, dosimetric (tolerance ≤ 2%) and geometric (tolerance ≤ 1 mm) end-to-end testing has been performed according to AAPM TG-142 [18], with special attention to hidden target tests for the detectability of the implanted markers used in our institution and the accuracy of marker positioning using phantoms. Submillimetre positioning accuracy is typical [19]. For quality assurance, a daily check lasting 5 min is required to check deviations from radiation isocentre and between the surface camera and XR positioning, and monthly thermal to 3D surface calibration as well as radiation isocentre calibration is performed if indicated.

Transrectal implantation of 1.2 mm x 3.0 mm 99.99% gold markers was performed with transrectal ultrasound guidance (TRUS) at least ten days before planning CT. At least three markers in a triangular pattern are required to enable calculation of both translational and rotational shifts. Two markers are placed into the base of the prostate, one on the left and one on the right side, and a third one in the apex only on one side of the organ. The goal is to achieve the largest possible spatial separation between the markers with a minimum desired distance of 2 cm to ensure accurate calculation of the 6D position, as well as to keep an adequate distance from the urethra thereby preventing unwanted injury.

**Fig. 1:**
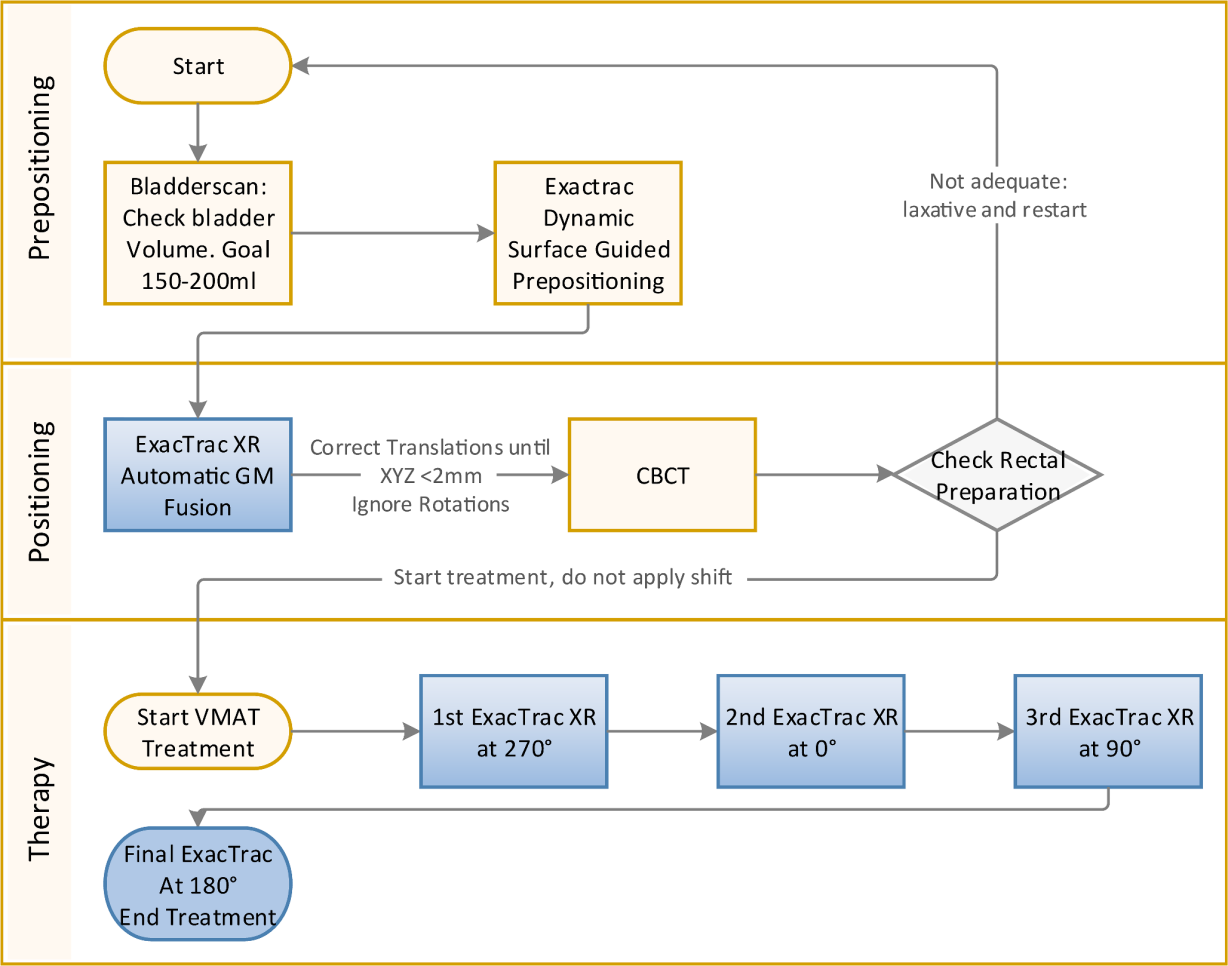
Summary of the treatment workflow

An overview of the treatment workflow is presented in Fig. 1. Before the start of each treatment session, adequate bladder filling was checked by ultrasound. Then the patient was prepositioned using surface guidance. Final positioning was performed using gold marker based XR guidance. A CBCT was performed to verify position and preparation of OARs (rectum and bladder) before treatment start. During the execution of the VMAT arc, intrafractional XR monitoring was performed three times (every quarter arc, at 270°, 0°, 90°), and a final XR position measurement was performed after completion of the treatment arc at 180°. If the translation tolerance of 2 mm on any axis was exceeded, the automatic beam-hold was engaged, and an intrafractional position correction was performed. Rotational errors were not corrected.

Data acquired for this study included the position deviation in x (lateral), y (longitudinal), and z (vertical) translation and x° (pitch), y° (roll) and z° (yaw) rotation, as well as the gantry angle of the measurement and the applied shift (if any). To assess the potential improvement in treatment accuracy through multiple intrafractional positional corrections according to the workflow, the measured deviations were recorded against a simulated control data set, with performed intrafractional corrections each being subtracted from the consecutive positional measurements. A paired sample T-test was used to compare overall corrected and uncorrected translational treatment accuracy. Time dependence was analysed using a one-way ANOVA, to compare measurements taken at different time points during the VMAT arcs. Significance level was set at p < 0.05. Statistical analysis was performed using SPSS statistics V27.0 (IBM Corporation, Armonk, NY, USA).

## Results

**Fig. 2:**
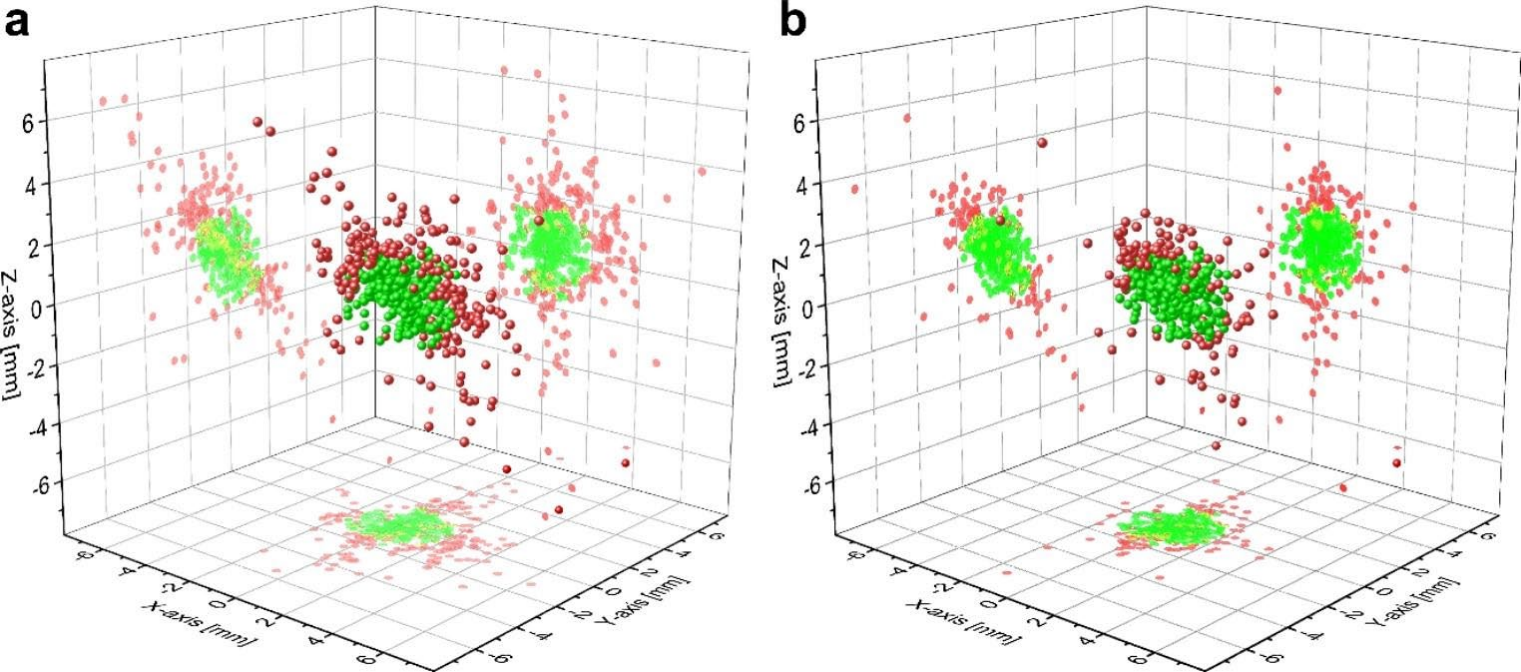
3D scatterplots of intrafractional translational isocentre deviations of the prostate. 3D position deviations of implanted fiducial markers without (**a**) and with (**b**) applied intrafractional correction are depicted. 3D deviations laying within the tolerance threshold are delineated as green dots, whereas red dots show deviations > 2 mm. Target movements without intrafractional corrections are calculated by eliminating the applied xyz-correction shifts during the course of each session from the actually recorded position data. Positional data of fiducial markers was acquired by repeated intrafractional measurements using the ExacTrac Dynamic system

From March to May 2022, 677 single XR measurements in 149 treatment sessions of 22 individual patients were included in this study. Without any intrafractional motion management, the frequency of motion shifts during treatment delivery exceeding the threshold of 2 mm was calculated to amount to 39.4%. By utilizing the EXTD system for intrafractional corrections, exceedance of the 3D-motion threshold was reduced to 20.6% of all measurements. By applying corrections, the mean intrafractional positioning precision was significantly improved at 1.39 (± 1.01 SD) mm versus 1.97 (± 1.44 SD) mm if motion management would not have been performed (p < 0.0001, see Table 1). The predominant prostate motion was detected in the anterior-superior and posterior-inferior direction (see Fig. 2).

**Table 1:** Summary of 3D deviation results with and without the use of intrafractional position corrections during IGRT of the prostate

3D deviation	EXTD correction	Ø EXTD correction
> 0.5 mm	83.7%	91.2%
> 1.0 mm	58.8%	74.8%
> 2.0 mm	20.6%	39.4%
> 3.0 mm	5.9%	16.4%
mean	1.39 mm	1.97 mm
SD	1.01	1.44

.

**Fig. 3:**
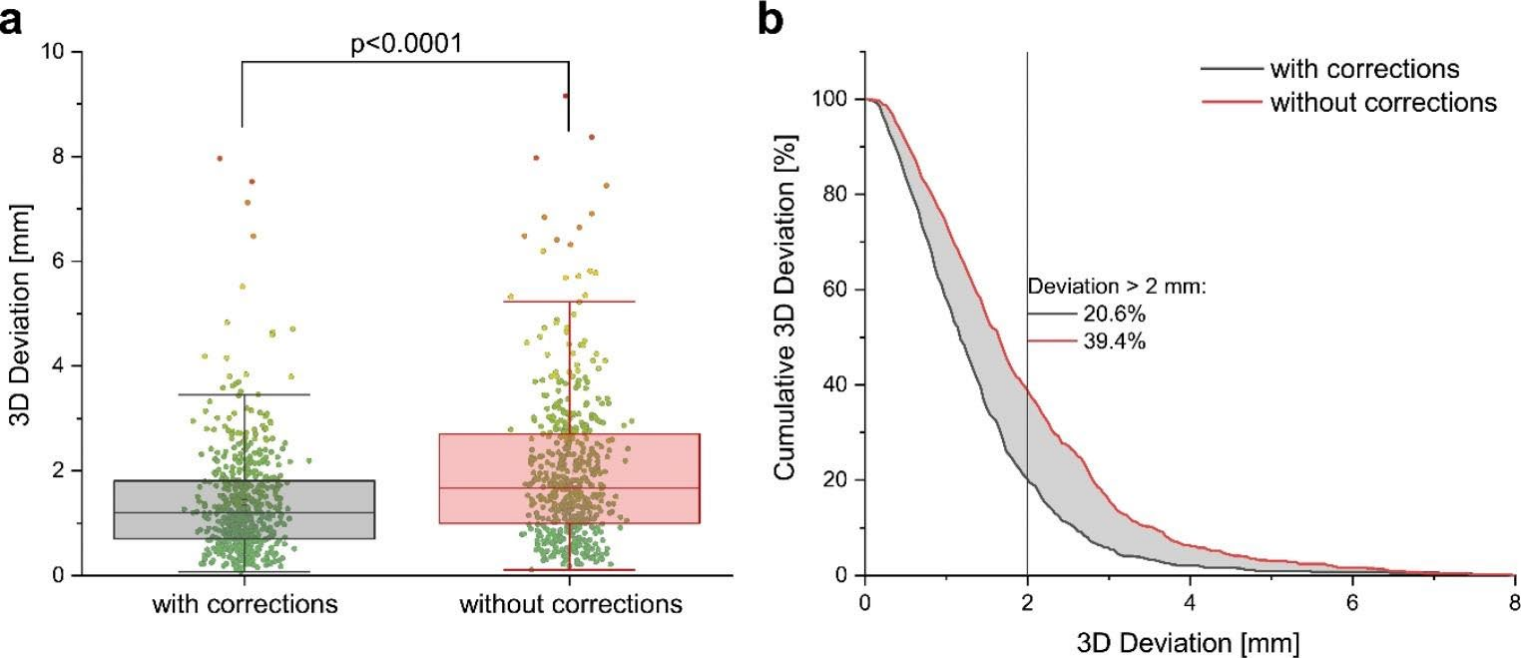
Effect of multiple intrafractional corrections on the magnitude of 3D deviations (3DV). **a**) Box plot of 3DV with and without applied corrections of prostate position during single treatment sessions. **b**) Cumulative histogram of 3DV distribution with and without performing corrections in prostate position

During 52 (34.9%) out of 149 treatment sessions, an intrafractional correction to compensate for prostate motion exceeding tolerance of > 2 mm in either the x, y, or z-axis was performed. Multiple corrections were necessary during nine (6.0%) sessions (see Fig. 3). Out of 539 intrafractional measurements, a 3D-deviation exceeding 1 mm, 2 mm, and 3 mm was detected in 317 (58.8%), 111 (20.6%), and 32 (5.9%) EXTD recordings, respectively (see Table 1). In comparison, in the control dataset without corrections, a 3D deviation larger than 1 mm, 2 mm, and 3 mm was observed in 404 (74.8%), 214 (39.4%), and 91 (16.4%) of measurements, respectively. If uncorrected, 15.9% of all recorded translational shifts larger the 2 mm re-entered the predefined margin within the subsequent measurement, whereas 84.1% were systematic shifts that remained out of tolerance for at least two consecutive measurements.

**Fig. 4:**
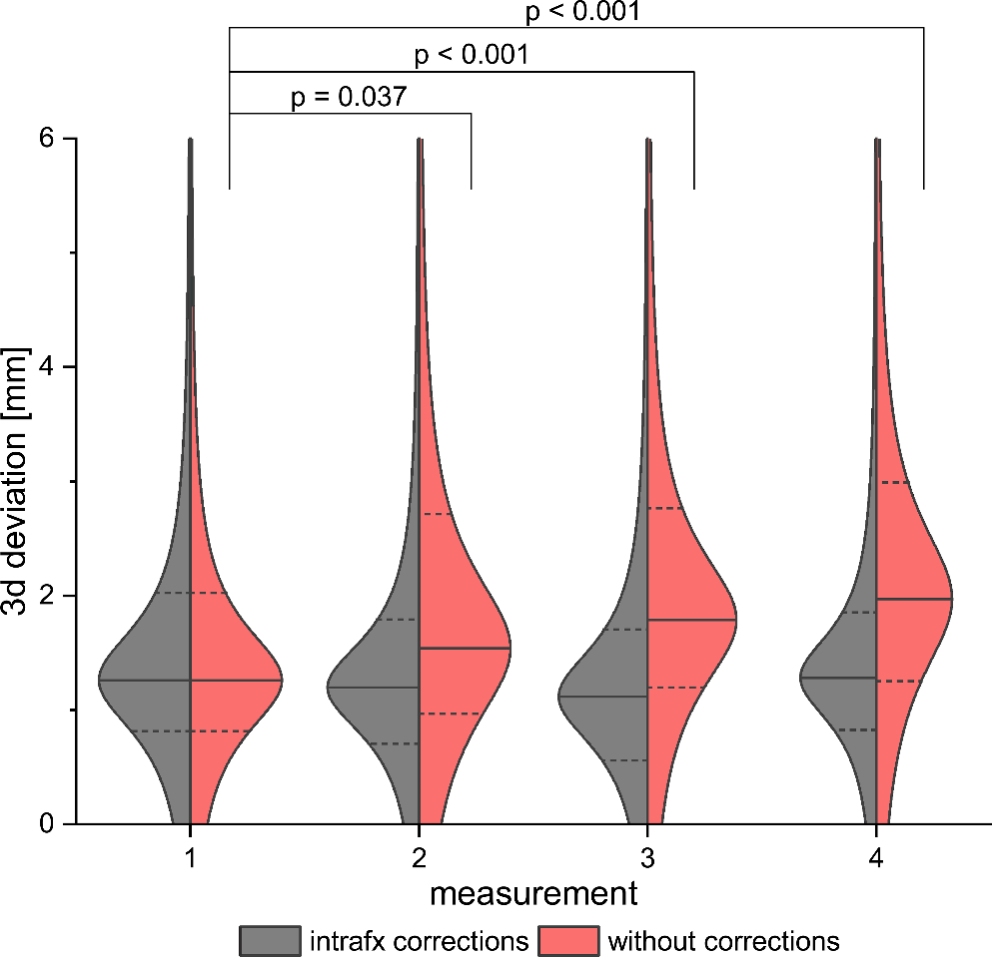
Distribution of intrafractional 3D deviation in prostate position at each time point during a VMAT arc. Violin plots depict the changing distribution pattern with and without the use of intrafractional position corrections. Intrafractional measurements were performed every quarter arc of IGRT.

A one-way ANOVA was performed to investigate whether there is a difference in 3D-deviation between the four position measurements taken during the VMAT arc. Accordingly, by applying intrafractional corrections no significant difference could be found (F = 0.963, p = 0.41). Without performing such corrections, a significant difference in mean 3D-deviation would have occurred (F = 11.30, p < 0.001). In addition, Tukey’s post hoc test for multiple comparisons revealed a significant difference between the first and all other measurements (p = 0.037, p < 0.001, p < 0.001), as well as between the second and fourth measurement (p = 0.028). The mean 3D-deviation rose in every single consecutive measurement of the dataset without EXT correction, from 1.45 mm at the first, up to 2.39 mm at the fourth measurement (see Fig. 4).

There was no statistically significant difference in positioning between patients treated with moderately hypofractionated and normofractionated treatment schedules, neither in overall accuracy (mean 3D deviation 2.12 mm vs. 1.92 mm, p = 0.136), nor between the individual measurement time points.

**Fig. 5:**
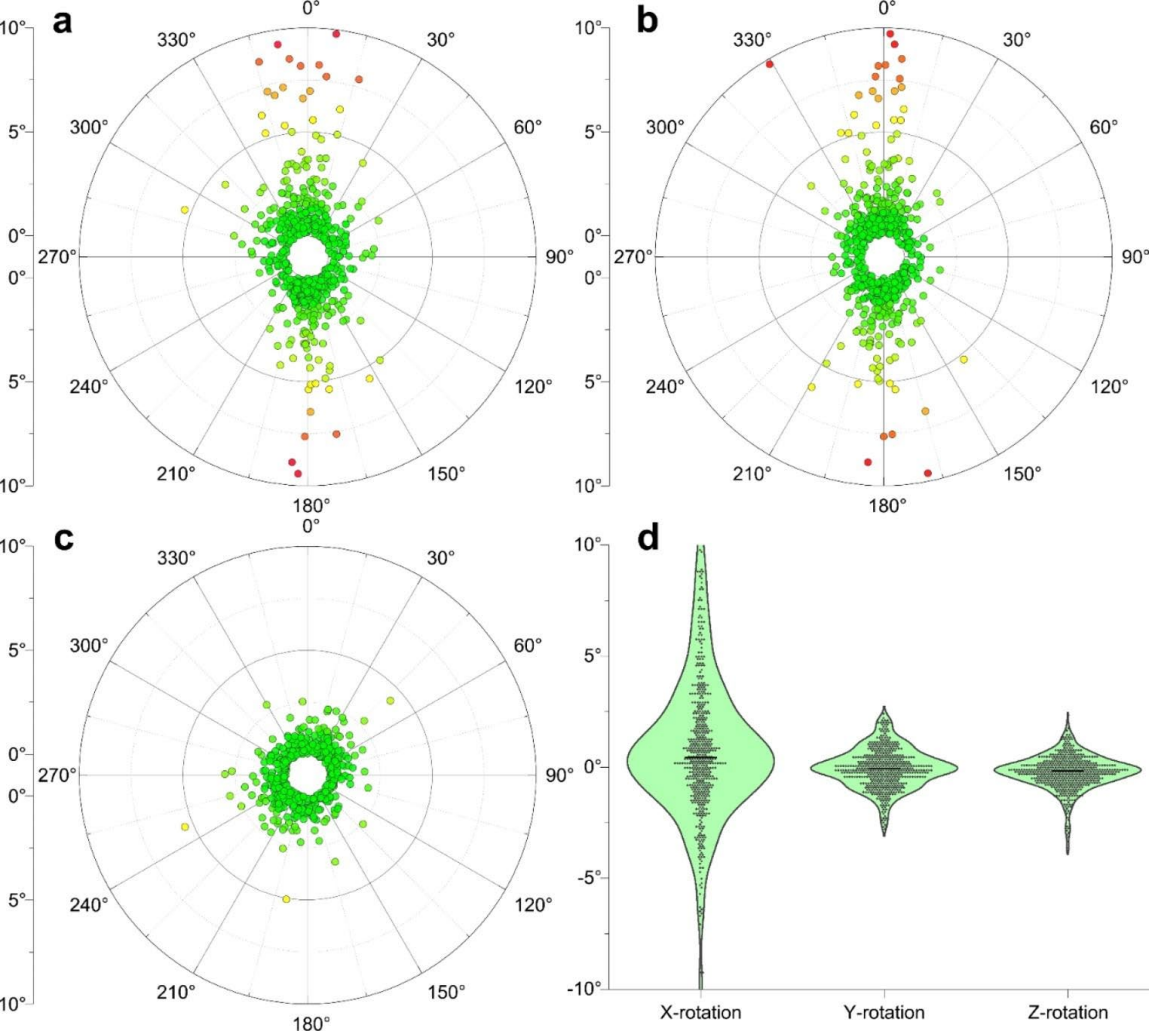
Polar graphs of intrafractional rotational isocentre-deviations of the prostate. Rotational deviations of implanted fiducial markers of **a**) x° (pitch) and y° (roll), **b**) x° and z° (yaw), and **c**) y° and z° are depicted. **d**) Distribution of angular deviation in pitch, roll and yaw. The largest angular deflections of the prostate position are observed in the pitch direction, whereas angular inaccuracies in the roll and yaw direction were minimal

**Fig. 6:**
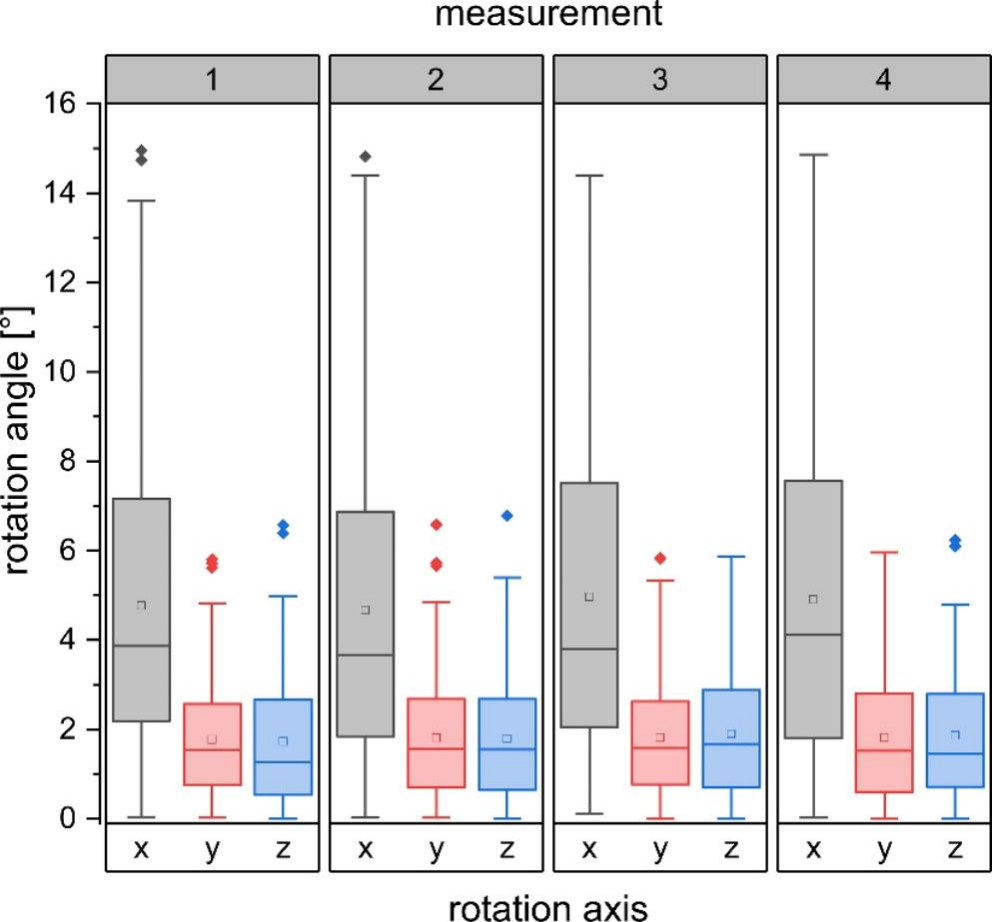
Magnitude of intrafractional 3D rotational errors in the pitch (x), roll (y), and yaw (z) direction at each time point during a VMAT arc displayed as box plots. Measurements were performed after each quarter arc

Using the EXTD-system, also rotational shifts of prostate positions in pitch (x°), roll (y°), and yaw (z°) were recorded along with every intrafractional 3D-position measurement (see Fig. 5). Of 677 EXTD measurements, recorded rotational shifts > 2° amounted to 217 (40.1%), 22 (4.1%), and 18 (3.3%) in the x°, y°, and z° directions, respectively. Angular deviations exceeding 5° were 68 (12.6%), 0 (0.0%), and 4 (0.7%) in the x°, y°, and z° directions, respectively. Systematic mean rotational shifts for x°, y°, and z° were + 0.83° (± 3.3 SD), − 0.03° (± 0.89 SD), and + 0.51° (± 0.77 SD), thereby reflecting a minimal systematic trend. In contrast to translational shifts, there was no significant difference in the extent of rotational errors from the first to the fourth measurement, neither in the pitch, the roll, nor the yaw axis (Fig. 6).

## Discussion

Moderate-, and more recently ultra-hypofractionated concepts for the primary treatment of prostate cancer have been demonstrated to offer equal outcome at a substantially reduced number of fractions [20–25]. However, the potential risk of increased toxicity [25], and the risk of target miss due to uncontrolled prostate motion [4] are still of concern. In evidence, the reduced number of fractions, and the effort to reduce the extent of safety margins to minimize toxicity make hypofractionated treatment schedules disproportionally more sensitive to prostate motion. Thus, monitoring for intrafractional motion can be considered a requirement for safe and effective implementation of prostate SBRT.

Depending on prostate localization and motion direction, inter- as well as intrafractional organ motions cause increased or decreased dose delivery to adjacent OARs. If unmonitored and uncorrected, dose benefits for rectum and bladder are reported to be inversely correlated, i.e. motions reducing dose coverage of one OAR may generally increase it in the other [12]. Thus, preventing excess dose delivery to either the bladder and/or the rectum can only be minimized by correction of prostate motion.

In this study, we analysed the intrafractional motion of the prostate by means of multiple XR-based measurements (ExacTrac-Dynamic) during each VMAT treatment-arc of 149 individual treatment sessions including 677 single measurements. The primary aim of the study was to evaluate the impact of intrafractional corrections on overall treatment accuracy. By applying intrafractional corrections, 3D-deviations exceeding 2 mm were limited to 20.6% of all quantified prostate motions. Without corrections, however, prostate 3D-motions larger than 2 mm would have been expected to an extent of 39.4%. Correspondingly, the mean intrafractional deviation was reduced from 1.97 (± 1.44) mm to 1.39 (± 1.01) mm. The measured intrafractional data on real-time translational prostate motion is in good agreement with previously reported observations [4, 26–28]. The recorded intrafractional rotational deviations were comparable in distribution and magnitude to analogous reports [29]. Rotational errors were however not corrected in this study. Rotational corrections are more challenging due to technical limitations, however, can be implemented using couch, MLC and collimator corrections as demonstrated in the literature [30–33]. Analysis of uncorrected rotational errors over treatment time did not reveal a significant increase (Fig. 6). This indicates that repeated intrafractional monitoring of rotational error might not add substantial benefit to treatment accuracy. In addition, because the shape of prostate target volumes are often highly spherical, the dosimetric difference due to uncorrected rotational errors are minimal. However, in case of non-spherical planning target volume (PTV) (i.e. in case of inclusion of seminal vesicles) a larger dosimetric impact has to be assumed [34]. More research is needed to clearly address the contribution of translational versus rotational displacements of the prostate to reduced dose coverage or excess OAR co-irradiation [29, 35], and especially to investigate feasible mitigation strategies for rotational errors, such as robust-optimized plans that account for rotational position shifts [36].

In general, an optimal setup would provide continuous non-invasive tracking and immediate continuous position adjustments during each SBRT session. So far, several monitoring systems utilizing different imaging methods have been developed, each exhibiting individual benefits and weaknesses in detecting and correcting intrafractional motion. These include kV, MV, MV-kV [37], or MR imaging techniques [38]. Commercial solutions include ExacTrac-Dynamic®, InTempo®-CyberKnife [39, 40], and Calypso® [12]. The most common monitoring application is based on implanted fiducial markers, which are suitable for intermittent position recordings. Transmitter or transponder-based systems allow for continuous tracking. Some define a catheter-based transmitter system (RayPilot® HypoCath) as less invasive [10]. However, repeated transmitter placement before each treatment session is required. The only system allowing for continuous and, in addition, marker-less tracking is MR monitoring [38]. It provides surrogate-free assessment of prostate and OAR positions as well as of real anatomic deformations. However, MRI-linacs are highly limited in availability and can be cost prohibitive. In conclusion, each solution to monitor organ motion targets to improve treatment accuracy. Thereby, the systems differ in patient comfort, necessity of invasive procedures, ease of use and set-up time, in compatibility with existing treatment and positioning devices, and last but not least, in costs and availability.

The kV-based ExacTrac-Dynamic system used in this study newly provides image-guided patient setup using implanted fiducial marker fusion, as well as, repeated intrafractional and fully automated marker-based recordings of prostate motion combined with continuous surface guidance. The solution is also suitable for a range of other IGRT applications necessitating XR or surface tracking. The application of the ExacTrac-Dynamic system discontinuously monitors prostate motion within single VMAT sessions, and it turned out not to increase positioning and treatment time. The implementation of the system was advantageous, since we detected significant prostate motion demanding correction during 34% of all monitored treatment sessions. Our analysis also confirmed previous reports [10, 41] on uncorrected 3D-deviations continuously increasing over the duration of a VMAT arc, whereas the accuracy can be maintained at the same level throughout the entire treatment session by performing intrafractional corrections. Since prostate motion can unpredictably occur at any time during a treatment session, the importance of intrafractional monitoring has to be emphasized not only for prolonged treatment durations (as is the case with cyberknife), but also during VMAT arc treatments lasting for few minutes only [12, 41]. In fact, for individual patients significant prostate displacements can occur even within this short time frame after initial positioning. In the context of SBRT, a single unobserved prostate motion could therefore potentially cause a relevant under-coverage of the target volume, potentially accompanied by excessive OAR toxicity. In evidence, we detected that if uncorrected, only 15.9% of all recorded translational shifts were transient and re-entered the predefined threshold margins (2 mm) within the subsequent measurement, whereas 84.1% were persisting systematic shifts. The observed increasing systematic shifts with prolonged treatment time from 1.45 mm at the first up to 2.39 mm at the last measurement (Fig. 4), is likely caused by a continuous build-up of randomly occurring motions. While the individual contribution of causes for organ movements is not well understood, changes in bladder filling, rectal distention, and muscle tension are known factors [42].

In a recent report of Panizza et al., intrafractional 3D motions exceeding a 2 mm tolerance were observed in 45% (25 of 56) of fractions during continuous monitoring. Position correction of deviations exceeding the tolerance for more than 15 s was necessary in 18% of sessions. Accordingly, acquiring new intrafractional CBCTs and re-referencing the used RayPilot® HypoCath system to the new position was required. The mean absolute deviation in the x, y, and z direction were reported to be 0.65 mm, 1.17 and 1.42 mm respectively. In comparison to Panizza et al., we used discontinuous intrafractional position monitoring (of implanted fiducial markers), with the advantage of receiving at least 3 documented x-ray images per arc without the need of additional image verification. Although the amount of recorded prostate shifts is, by nature, lower following discontinuous marker detection (i.e. 34.0%; 52 out of 149 sessions), corrections could be performed after every recorded displacement within seconds, thereby not impacting overall treatment duration. Using discontinuous tracking, the risk of missing small displacements occurring between individual measurements of course persists. With our setup, a quarter arc could be delivered in the worst case, before the deviation is registered by the next EXTD measurement. However, if considering the necessity of mounting a catheter before each treatment session, continuous monitoring by using transponders clearly exceeds the treatment time of implanted marker-based applications. Since the frequency and extent of prostate movements increase with increasing treatment duration, repeated intrafractional individual measurements with the EXTD system ensure at least a faster treatment process, which has been proven to result in a lower probability of significant target shifts. For physicians it therefore remains a matter of concern, whether detecting every intrafractional short-term prostate displacement outweighs a substantially increased patient positioning and treatment effort.

Another possibility for providing intrafractional IGRT is the utilization of the on-board kV CBCT to acquire 2D fluoroscopic imaging [43, 44]. Such a solution can be used for continuous tracking of fiducial markers, and does not require the installation of additional equipment, since CBCT is commonly integrated in modern LINACs. However, this method is not capable of directly acquiring 3D position information, instead relaying on motion prediction algorithms to estimate the real 3D position of the target, which by nature cannot compete in accuracy with 3D imaging methods. Moreover, in case of exceeded tolerance, reacquiring a pair of kV images with 45° or 90° separation is needed in order to calculate the offset to be then used for position correction. In contrast, the EXDT system directly provides 3D position information, which, if out of tolerance, can directly be used for immediate correction without any additional imaging required. An advantage of 2D fluoroscopic imaging is the (near) continuous tracking of prostate motion [44], whereas the EXDT is limited by the ability to acquire measurements at certain gantry angles only, due to the obstruction of the two panels by the gantry. However, also with fluoroscopic imaging, the accuracy is reduced at certain angle gantry ranges due to the obscuring of the seeds on the kV images by bony structures [43].

Due to the prolonged session times in hypofractionated treatments, the impact of prostate motion is disproportionally higher than compared to conventionally fractionated treatments. As corroborated by previous studies positioning accuracy decreases with treatment duration, which has a direct impact on OAR toxicity and target dose coverage. Due to the reduced number of fractions, single organ movements have a disproportionate impact on the overall accuracy and effectiveness of the therapy. Therefore, the benefit of performing intrafractional monitoring becomes more evident with increasing single fraction doses in hypofractionation, and can be considered a necessity for safely performing SBRT. The system utilized in this study is automatically working in parallel to treatment and acquisition of intrafractional prostate position monitoring does not require additional effort. In the case of a required beam-hold and subsequent positioning correction, a mean of 30.4 s (per correction) had to be added to the total treatment-time. Considering the total need of repositioning after detection of threshold-exceeding shifts, which was the case for 20.6% of all sessions, this result implicates that each VMAT arc is extended for 6.3 s on average. The impact of performing intrafractional IGRT on the time management for the daily clinical routine is thus negligible. Thus, it could also be considered for application in conventional fractionation for additional patient safety. An additional clinical benefit for conventional fractionation is however expected to be less pronounced, given the shorter treatment times and higher session count.

With the routine implementation of intrafractional position monitoring and immediate corrections, reduction of PTV margins while maintaining target coverage is conceivable, thus providing the opportunity to increase the therapeutic window in SBRT of prostate cancer. Other sources of errors must however be taken into account when determining optimal PTV margins. These include errors derived from the treatment preparation process, such as image acquisition inaccuracies, fusion errors, target delineation inaccuracies, as well as dosimetry and machine-derived inaccuracies. In addition, intrafractional monitoring of fiducial marker position cannot replace a volumetric IGRT system for initial positioning, since optimal rectal and bladder filling must also be assured.

## Conclusion

Intrafractional anatomic changes represent a significant source of treatment uncertainties endangering safe and effective application of hypofractionated dose concepts in prostate cancer. Intrafractional surveillance of organ motion can prevent insufficient target coverage as well as excessive co-irradiation of OARs. We could demonstrate, that repeated XR-imaging of implanted gold fiducial markers recording translational displacements led to position corrections in more than one third of all VMAT treatment sessions. Thus, we clearly recommend applying repeated or continuous intrafractional monitoring of prostate position especially when performing ultra-hypofractionated treatment concepts.

### Electronic supplementary material

Supplementary figure
