# Supplementary material for: Dynamic intrafractional position monitoring with implanted fiducial markers for enhanced accuracy in radiotherapy of prostate cancer
Source: Phys Eng Sci Med. 2023 Jul 31;46(4):1365–74. doi: 10.1007/s13246-023-01304-w (PMC10703977; doi:10.1007/s13246-023-01304-w)
Supplement: Supplementary file 1 — Supplementary figure [file 13246_2023_1304_MOESM1_ESM.doc]

# **Supplementary Material**

# **Dynamic intrafractional position monitoring with implanted fiducal markers for enhanced accuracy in radiotherapy of prostate cancer**

**Authors:** Julian Mangesius (1), Thomas Seppi (1), Ramine Ibrahim (1), Katrin Fleischmann (1), Angela Ginestet (1), Samuel Vorbach (1), Tilmann Hart (1), Martin Pointner (1), Stephanie Mangesius (2), Ute Ganswindt (1)

(1) Department of Radiation Oncology, Medical University of Innsbruck, Innsbruck, Austria
(2) Department of Neuroradiology, Medical University of Innsbruck, Austria


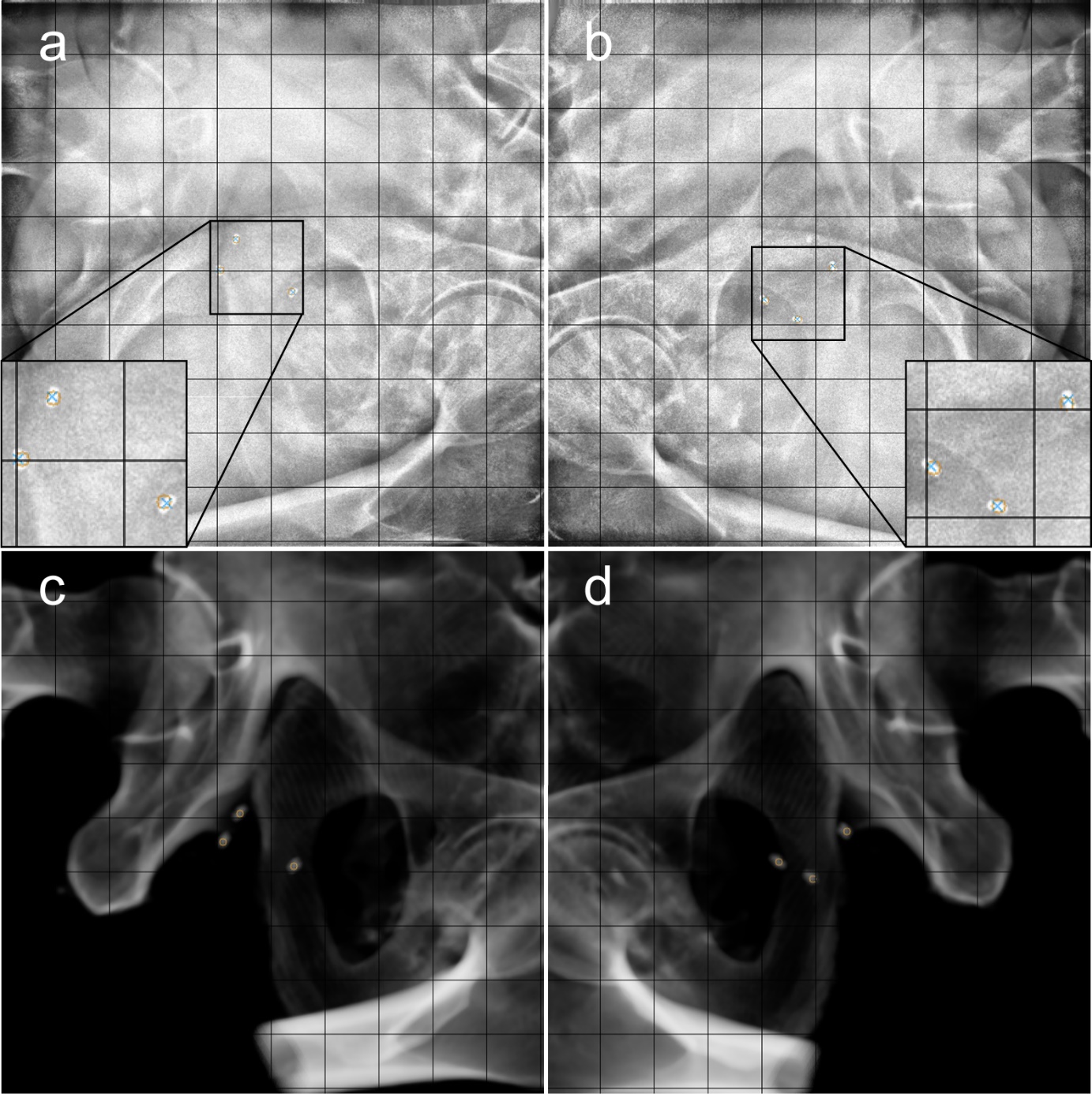


**Supplementary fig. 1** Images acquired by the **a)** left and **b)** right ExacTrac Dynamic XR tubes (120 kV, 20mAs). The three implanted fiducial markers are visible and detected automatically by the system (magnified 2x). Corresponding digital reconstructed radiographs (DRR) generated from the planning CT are depicted in panel **c)** and **d)**.
